# Mechanisms of Myofibre Death in Muscular Dystrophies: The Emergence of the Regulated Forms of Necrosis in Myology

**DOI:** 10.3390/ijms24010362

**Published:** 2022-12-26

**Authors:** Maximilien Bencze

**Affiliations:** 1 “Biology of the Neuromuscular System” Team, Institut Mondor de Recherche Biomédicale (IMRB), University Paris-Est Créteil, INSERM, U955 IMRB, 94010 Créteil, France; maximilien.bencze@inserm.fr; 2 École Nationale Vétérinaire d’Alfort, IMRB, 94700 Maisons-Alfort, France

**Keywords:** muscular dystrophies, duchenne muscular dystrophy, myonecrosis, myofibre, regulated cell death, regulated necrosis, necroptosis, apoptosis

## Abstract

Myofibre necrosis is a central pathogenic process in muscular dystrophies (MD). As post-lesional regeneration cannot fully compensate for chronic myofibre loss, interstitial tissue accumulates and impairs muscle function. Muscle regeneration has been extensively studied over the last decades, however, the pathway(s) controlling muscle necrosis remains largely unknown. The recent discovery of several regulated cell death (RCD) pathways with necrotic morphology challenged the dogma of necrosis as an uncontrolled process, opening interesting perspectives for many degenerative disorders. In this review, we focus on how cell death affects myofibres in MDs, integrating the latest research in the cell death field, with specific emphasis on Duchenne muscular dystrophy, the best-known and most common hereditary MD. The role of regulated forms of necrosis in myology is still in its infancy but there is increasing evidence that necroptosis, a genetically programmed form of necrosis, is involved in muscle degenerating disorders. The existence of apoptosis in myofibre demise will be questioned, while other forms of non-apoptotic RCDs may also have a role in myonecrosis, illustrating the complexity and possibly the heterogeneity of the cell death pathways in muscle degenerating conditions.

## 1. Introduction

The skeletal muscle is the largest tissue of the human body, representing 30 to 40% of the body mass. Beyond its crucial function in locomotion, it also has important roles in body homeostasis, such as the regulation of body temperature and hormonal activity [1]. Skeletal muscle is mainly constituted of gigantic and elongated plurinucleated cells called muscle fibres (myofibres) with contractile properties. Hundreds of nuclei (myonuclei) can be included within the cytosol of mature myofibres, also called sarcoplasm. Myonuclei are typically located by the plasma membrane, at the periphery of the myofibre. The plasma membrane of mature myofibres is often named sarcolemma as it refers to the association of the myofibre plasma membrane and associated proteins that have a central role in skeletal muscle homeostasis. Control of myofibre contraction is assured by terminal axons from motor neurons at specialised synapses called neuromuscular junctions. Presynaptic acetylcholine release and subsequent binding to nicotinic receptors trigger myofibre contraction and a reduction of cell length, which distributes the mechanical force along the sarcolemma. Tension is transmitted to the extracellular matrix thanks to the sarcolemmal dystrophin-associated protein complex (DAPC) which plays a crucial structural role and a resistance to mechanical stretch. By linking the actin cytoskeleton to the extracellular matrix, DAPC proteins stabilize myofibres during contraction and prevent tension-elicited plasma membrane breakdown [2]. However, acute or traumatic exercise can dramatically compromise the physical integrity of sarcolemma causing leakage of sarcoplasm content into the extracellular compartment. After myofibre injury, sarcoplasm leakage is followed by the ultimate and definitive contraction of myofibres and cell degeneration.

The strong regenerative capacity of skeletal muscle tissue is due to resident muscle stem cells (MuSCs), also called “satellite cells”. MuSCs are usually found in a quiescent state, beside myofibers and underneath the basal lamina of myofibres basal lamina [3]. Myofibre injury activates MuSCs to commit the myogenic lineage, which is under the control of several transcription factors such as PAX7 and Myogenic regulatory factors (MRFs). MRFs control myogenesis by guiding MuSCs into phases of proliferation, myogenic commitment, differentiation, and fusion to form newly formed myofibres. In parallel, a subpopulation of proliferating MuSCs participates in the renewal of a pool of quiescent MuSCs by relocating into satellite cell niches so that further regenerative events can be achieved in case of injury (For review, see [4]).

Following muscle damage, the infiltration of inflammatory cells such as neutrophils and macrophages, although promote injury, can initiate a recovery in damaged muscle cells through tissue healing [5,6]. Muscle regeneration is tightly controlled by the evolution of the inflammatory environment by controlling MuSCs fate [7]. Despite their cytotoxic profile, classically activated subpopulations of macrophages (M1) support myogenesis by promoting MuSC proliferation and delaying the myogenic commitment of MuSCs resulting in cell migration within the injured area [8]. The phagocytic activity of dead cells debris by M1 pro-inflammatory macrophages generates a phenotypic switch towards an anti-inflammatory profile (M2) around 4–5 days post-injury which is required for wound healing by promoting MuSC differentiation and fusion. Furthermore, studies show that depletion of inflammatory cells can impair muscle healing in vivo [7,8]. Surprisingly, the molecular basis of muscle regeneration is much better understood than the cell death process of myofibres, regardless of the nature of the insult. Muscle damage involving myofibre demise comes with necrotic features in physical or chemical insults, snake venom-mediated injury, or pathological lesions [9,10,11,12]. It is also accompanied by typical cell death mediators such as Ca^2+^ overload, energetic crisis, ROS generation, osmotic swelling, releasing sarcoplasmic content and triggering inflammatory infiltration [13,14,15].

Until recently, cell necrosis was described as an unregulated process, i.e., executed at the intracellular level without the requirement of a proper signalling pathway. However, the identification of non-apoptotic types of regulated cell death pathways leading to necrotic morphology (also mentioned as regulated necrosis) provided a breakthrough in our understanding of the pathogenesis of degenerative disorders. Recently, regulated necrosis (RN) has been identified in the pathogenesis of conditions affecting multiple organs with specific emphasis on neuroscience, cardiovascular research and cancer [16]. Only in 2018 did the role of RN start to be addressed in myology [17,18,19,20], opening new perspectives for research and clinical strategy in degenerative neuromuscular disorders.

This review will focus on describing the molecular mechanisms underlying the cell death process of myofibre loss in muscular dystrophies, with specific emphasis on Duchenne muscular dystrophy. Recent advances in the field of cell death research will be summarized, and how this may profoundly improve our understanding of myofibre loss in degenerative neuromuscular disorders (NMD).

## 2. Myofibre Death in Muscular Dystrophies

Neuromuscular disorders (NMD) form a highly heterogeneous family of conditions impairing muscle function. The classification of NMDs integrates different parameters, and most specifically the nature of the cell types showing a phenotypic default. NMD can originate from cellular dysfunctions affecting either muscle or neural cells. Myopathies are inherited or acquired rare diseases characterized by myofibre dysfunction which is independent of any defaults in the peripheral central nervous system. Muscular dystrophies (MDs) are defined as a subgroup of inherited myopathies with progressive muscle weakness due to muscle degenerating events. A large proportion of MDs is caused by variants affecting a protein belonging to the DAPC complex of the myofibre sarcolemma. This includes mutations in genes coding for dystrophin in Duchenne and Becker muscular dystrophy (respectively DMD and BMD), proteins α-, ß-, δ-, γ- and ε-sarcoglycan proteins, dystroglycan, and α-2 laminin. Within non-sarcolemmal MDs, the best-known conditions involve the mutations affecting other proteins such as DMPK in Myotonic dystrophy type 1 and DUX4 in facio-scapulo-humeral muscular dystrophy (FSHD) [21].

Typical features of muscle biopsies from MD patients show myofibre degeneration with necrotic morphology, termed “myonecrosis” [22]. Direct consequences of leaky sarcolemma myonecrosis include an increase of creatine kinase (CK) release in the blood (hyperCKmia), the uptake of proteins belonging to the blood compartment into leaky myofibres [23], associated with typical inflammatory consequences of necrotic demise. This includes the infiltration of dead myofibres by phagocytic cells (necroinflammation), followed by muscle regeneration [24]. Reactive oxygen species (ROS) are often associated with myofibre death [25,26], even if their precise role in pathogenesis remains unclear as ROS can be generated by cells submitted to sublethal or lethal stress [27]. An indirect consequence of chronic degeneration is the incapacity of myogenesis to fully compensate for myofibre loss, by accumulating scar tissue such as fibrosis and adipocytes, and participating in the loss of the contractile function [28]. Dystrophic muscles also show an increased proportion of unusual atrophic or hypertrophic myofibres compared to healthy muscles, as a consequence of the significant remodelling process [21,22].

### 2.1. Myofibre Death in Dystrophinopathies

Dystrophin is a cytoskeletal protein encoded by the *DMD* gene which is a key component of the DAPC complex. The DMD gene, located at the X chromosome is particularly long (2.2 Mb) and 99% constituted by introns. Seven tissue-specific promoters encode three full-length isoforms, fragmented in 79 exons [29]. There are several thousand identified variants in dystrophin [30]. However, about 70% of DMD patients show a mutation in the genomic hot spot region located between exons 45 and 53 [31,32]. According to the nature and location of the mutation, the reading frame can be disrupted. The complete absence of dystrophin expression will typically generate a severe phenotype (DMD). Variants allowing the translation of a truncated protein expressing the actin and dystroglycan binding domains generate a late-onset and milder phenotype, known as Becker muscular dystrophy) [32]. DMD is the most common muscular dystrophy in infants and the most extensively investigated. Initially DMD young boys typically show a dramatic increase in blood CK levels associated with a slight delay in motor development. After 3–4 years, muscular weakness worsens and DMD boys develop a pseudo-hypertrophy of the calves. By thirteen years old, patients lose their autonomous walking ability, step by step giving raise to increasing respiratory failure and the development of cardiomyopathy, with deterioration of speech and swallowing [33,34]. There is no cure for DMD, and patients typically die from cardiorespiratory failure.

In line with what is observed in human patients [35], animal models of DMD lacking dystrophin are affected by the necrotic fate in myofibres. These include mouse [36,37], dog [38], pig [39], rabbit [40], and rat [41,42,43] species. Interestingly, dystrophin deficiency does not trigger myofibre death per se, but it confers an important source of instability for cell homeostasis in vivo. Furthermore, given the scaffolding role of Dystrophin in myofibres, a greater susceptibility to mechanical stress during contractions occurs [2], which is exacerbated in eccentric contractions [44,45]. It also affects nitric oxide (NO) synthase expression and localization [46], indirectly playing roles in intracellular signalling of dystrophic myofibre (for review, see [47]). For example, abnormal Ca^2+^ influx has been extensively investigated in dystrophin-deficient myotubes [48].

A large variety of factors are associated with myonecrosis, including abnormal calcium handling, reactive oxygen species (ROS) and inflammatory molecules in dystrophin-deficient muscles [49,50,51]. However, whether some of these cytotoxic factors do actively promote myofibre demise or stand for biochemical consequences of cell stress is not always clear. As post-injury inflammation has been associated with a known cytotoxic effect, a detrimental role of inflammatory environment on myofiber survival has been hypothesized. From the 1970s, glucocorticoids (GCs) treatments have been used in cohorts of DMD patients generating promising results [52]. GCs, by intermittent and daily intake, are so far the only pharmacological treatment which provide significant improvement in skeletal muscle weakness and slows down the course of pathogenesis [53]. This includes benefits in muscle strength [54], prolonged walking capacity [55], and delayed respiratory insufficiency [33,34]. Beyond a reduction of myonecrosis [56,57,58], the complete mechanisms of action of GC remain elusive. Other properties of GC treatment on muscle cells have been highlighted and may also contribute to the improvement of the dystrophic phenotype [59,60,61].

Several inflammatory cytokines such as TNFα, IFNγ, and TNF-like weak inducer of apoptosis (TWEAK) are ligands to the tumour necrosis factor receptor (TNFR) family and are upregulated in dystrophin-deficient muscles [62]. TNFR superfamily is divided into two categories: activating and death receptors (respectively AR and DR). For instance, TNFα can bind to either TNFR1 (DR) or TNFR2 (AR). Whilst binding to TNFR2 can activate nuclear factor κB (NF-κB) and mitogen-activated protein kinase (MAPK) pathways, TNFR1 is both AR and DR, therefore potentially activating both signalling pathways. As a consequence, TNFα can control cell proliferation, differentiation, or metabolism, either positively or negatively, and trigger cell death, depending on the cellular context which allows the balance between pro-death and pro-survival machinery [63]. NFκB and p38 MAPK signalling are largely described as regulators of myogenesis. In a dose-dependent manner, TNFα is required for MuSC differentiation via p38 MAPK pathway [64], while it prevents differentiation and promotes atrophy via NFκB activation [65]. TNFα has also been identified as mitogenic for MuSC in vitro and in vivo [66,67]. Interestingly, blocking TNFα binding dampens myofibre necrosis in mdx mice. TNF-elicited cell death is particularly efficient at the onset of mdx pathogenesis, when muscle degeneration is at its highest [68,69].

The involvement of other death ligands in mdx pathogenesis has also been addressed in DMD muscle wasting. Indeed, RG Korneluk and colleagues elegantly showed that cellular inhibitor of apoptosis (cIAP)1 depletion reduces overall pathology, including myonecrosis in some muscle groups of mdx mice such as the soleus and diaphragm [70]. However, this study does not specifically tackle the phenomenon of cell demise or identify a death ligand nor the cell death pathway responsible for cIAP1-mediated cell death. The same authors described that TNF-like weak inducer of apoptosis (TWEAK) binds to its receptor Fn14, which activates the non-canonical pathway of NFκB, leading to elevated NFκB inducing kinase (NIK) levels via cellular inhibitor of apoptosis 1 (cIAP1) protein in muscle. However, the proposed mechanisms of action seem independent of cell death induction and are suspected to specifically affect MuSCs behaviour and their regenerative capacity following cell demise [71,72].

IFNγ, a powerful pro-inflammatory cytokine produced by CD4+ T cells and natural killer cells, can efficiently prime macrophages to classical activation and promote a cytotoxic M1 phenotype. IFNγ is also upregulated in degenerating mdx muscles [73,74], thus it was originally hypothesized that IFNγ could actively take part in myonecrosis, either directly or indirectly via its promotion of a pro-inflammatory environment in muscle. The null mutation of IFNγ does indeed ameliorate muscle function in mdx mice without preventing myofibre death or necroinflammation [75], suggesting an overall detrimental role of IFNγ in the myogenesis of mdx muscle. IFNγ stimulation represses MuSC fusion in vitro [76], and in vivo, its upregulation in wild-type mice has been shown to delay proliferation, differentiation, and fusion myogenesis after injury and to promote fibrosis [77]. In these studies, no link with direct or indirect IFNγ-dependent cell death, regardless of the cell death mechanism, has been reported. A recent article reported that IFNγ sensitizes macrophages to toll-like receptor (TLR)-elicited cell death, requiring Caspase-8 and mitochondrial apoptotic effectors BAX and BAK. Interestingly, mdxTLR4^−/−^ mice present a reduced diaphragm pathology, associated with histological evidence of reduced inflammatory infiltration and central nucleation [78]. Here again, the authors report a cellular mechanism involving a shift from cytotoxic M1 phenotype to M2 phenotype to support their results, but direct evidence of a TLR4-mediated myofibre death is missing.

A hallmark of dystrophin-deficient cells is the dysregulation of calcium homeostasis and an increase of cytosolic calcium levels, with consequences on the activation of protease systems such as calpains [13,79]. Dysregulation of calpains activation correlates with disease extent (see review [80]). DNA fragmentation and positivity to TUNEL staining are often spotted in distinct species affected by dystrophin deficiency from C. elegans to humans [81,82,83,84,85].

### 2.2. Sarcoglycanopathies

Sarcoglycanopathies are autosomal recessive muscular dystrophies affecting the limb-girdle muscles (LGMD). They are caused by mutations in some genes coding for sarcoglycan (SG) proteins: α-, β-, γ- and δ-SGs are transmembrane glycoproteins belonging to the DAPC. Similar to dystrophin, sarcoglycans are crucial for the stability of the sarcolemma, protecting striated and cardiac muscle fibres from damage induced by muscle contraction [86]. LGMD involves the impairment of pelvic and scapular girdle muscles and the trunk, with varying degrees of cardiorespiratory defects. Pseudohypertrophy of the calves and tongue are often observed. Typically, very high CK levels are detected when one of the sarcoglycans is depleted, mimicking sever forms of DMD in many aspects and BMD in less affected patients [87,88]. Respiratory complications can be observed in around 25% of LGMD patients, and dilated cardiomyopathy is not rare in patients with variants in β-SG and γ-SG. Animal models lacking α-, ß-, δ-, and γ-SG have elevated CK levels, myonecrosis in limb and respiratory muscles, cardiomyopathy, and are associated with typical necrosis issues such as inflammation, fibrosis and myogenesis [89,90,91,92,93].

Little is known about the disease mechanism involved in the pathogenic course of sarcoglycanopathies. Despite knowledge of gene variants involved, the main putative cause of myofibre death of SG-deficient muscle is the loss of membrane integrity caused by mechanical stress in the context of DAPC default [91]. The stretching of γ-SG^−/−^ muscle cells dramatically stimulates the phosphorylation of p38 MAPK (T180/Y182) and ERK1/2 (T202/Y204) and their activation, indicating a hypersensitivity of mutated cells to signalling pathways induced by mechanical stress [94]. γ-SG deficiency has an impact on myofibre stiffness and time of relaxation after contraction. Regardless of mechanical stress, the intensity of TUNEL cell death assays is spontaneously upregulated in γ-SG KO myotube compared to WT [94], indicating DNA fragmentation and apoptotic-like cell death features. Calcium channel dysregulation has also been assessed, as in dystrophinopathies. More specifically, Ca^2+^ influx is dramatically increased in Bio14.6 myotubes from δ-SG-deficient hamsters with basal increased opening probabilities and decreased closed times [95], which is mediated by transient receptor potential cation channel subfamily V, member 2 (TRPV2) [96]. Necroinflammation is significant in sarcoglycanopathies [21]. Beyond the role of SG in the integrity of sarcolemma, SG exerts an ecto-ATPase activity which is depleted in SG deficient cells, dependent on the P2X7 purinoreceptor [97,98].

### 2.3. Dysferlinopathies

Dysferlin is a calcium-interacting protein belonging to the Ferlin family, which is involved in vesicle fusion. Its expression is required for myofibre membrane repair by sealing sublethal breaks in the myofibre plasma membrane. As skeletal muscle is subject to mechanical stretch during contractions, the role of dysferlin is particularly important and its absence makes myofibre prone to membrane leakage and major disturbances in cellular homeostasis. In humans, dysferlin mutations can lead to different forms of MD including Miyoshi myopathy and Limb-Girdle Muscular Dystrophy type 2B [99]. In line with previously mentioned MDs, dysferlinopathy is characterized by muscle degenerating features and inflammatory infiltrate [100,101]. Monocytes also express dysferlin and dysferlin-deficient macrophage enhance phagocytotic activity and upregulate inflammasome formation [102,103,104]. This suggests that the pathogenesis of dysferlinopathy involves muscle-independent factors such as the regulation of innate inflammatory response. Dysferlin is cytoprotective against TNFR1/2-dependant ischemic injuries [105]. While dysferlin is known to prevent apoptosis elicited by ischemia-reperfusion injuries [106], there is no clear evidence of apoptotic demise in dysferlinopathy. In contrast with previously mentioned MDs, dysferlin deficiency is not characterized by important necroinflammation, even if there is no specific evidence for non-immunogenic apoptotic demise [107].

### 2.4. Merosinopathy

Congenital muscular dystrophy caused by merosin/laminin α2 chain deficiency (MDC1A) is due to a mutation in the gene coding for the α2 subunit of the Laminin gene (LAMA-2). There are hundreds of identified variants resulting in a large clinical heterogeneity [108]. LAMA-2 deficiency leads to one of the most severe muscular dystrophies with dramatically limited life expectancy [21]. MDC1A patients rarely develop the ablity to walk. Elevated creatine kinase levels, inflammatory infiltrates, connective tissue proliferation and irregular myofibres size also characterize merosinopathy [109,110]. There are several mouse models for LAMA2, with various kinetics and extent of myonecrosis, all culminating with mouse lethality [111]. Necroinflammation and ROS are abundant in muscles from *Lam*a2^−/−^ mice, and antioxidative strategy reduced pathology in dy^2J^/dy^2J^ mice [112]. *Lama2*^−/−^ mice depleted for BAX, or overexpressing BCL-2 improved the pathology [113] suggesting a role for mitochondrial outer membrane permeabilisation (MOMP) to mediate cell death.

## 3. The Emergence of Non-Apoptotic Regulated Cell Death Pathways: A Conceptual Revolution

Over the last decade, an exciting new era has commenced in cell death focused research with the identification of necrotic subtypes of regulated cell death pathways. Several assumptions and dogma have then been profoundly challenged, and it appears many terms or concepts have been misused [114,115]. The field is rapidly evolving, with the continual discovery of new forms of cell death and updates of importance [116,117,118]. These breakthroughs have a significant impact on the understanding of the cellular mechanisms involved in a myriad of pathologies, affecting all organs, including skeletal muscle.

### 3.1. Defining Cell Death and the Different Cell Death Routes

Misunderstandings in cell degeneration often can be due to the obscure definition of cell death, with potential confusion with sublethal cell stress responses. Dying cells are defined by trespassing a point of no return where cell homeostasis is no longer compatible with survival. This process is therefore irreversible [114]. However, caution is required when interpretating data, especially when using markers (or ‘presumed markers’) of cell death. For instance, cleaved caspase-3 is the archetype of markers for apoptosis. However, observing caspase-3 activation in an injured tissue could be misleading, since caspase proteases are involved in the differentiation of multiple cell types in metazoans, such as in skeletal muscle [119,120,121]. Similarly, membrane phosphatidylserine (PS) exposure or MOMP are often considered a marker of apoptotic cell death. However, cell recovery after such events demonstrates that these events are not necessarily points of no return, and therefore do not represent death markers [122,123].

In 1964, the concept of “programmed cell death” was first stated in insect metamorphosis and then described in its genetic nature in C. Elegans [124,125] In the 1970s, a cornerstone element of the discipline was the classification of different types of cell death according to morphological discriminating parameters. Three distinct morphologies of cell death following exposure to toxins have been described in vivo: type I cell death was associated with heterophagy, type II cell death was associated with autophagy, and type III cell death did not involve digestion by other cells. These terms are no longer used and are replaced by apoptosis, autophagy and necrosis respectively [14,115]. To this cornerstone morphological criteria, biochemical routes have also been used to refine cell death classification.

### 3.2. Intrinsic and Extrinsic Apoptotic Pathways

Apoptosis has long been opposed to necrosis, particularly its consequences on tissue homeostasis. Cardinal features of apoptotic morphology include a round shape, extensive nuclear and organelle digestion, chromatin condensation, DNA fragmentation and plasma membrane rupture occurring at a very late step if the cells remaining are not already handled by phagocytic cells via efferocytosis [126]. The lack of plasma membrane leakage in apoptosis is illustrated by an absence of vital dye uptake such as propidium iodide (PI) and faint immunogenic consequences. On the contrary, necrotic features include an earlier step targeting plasma membrane integrity, and a massive release of cytoplasmic content, including damage-associated molecular patterns (DAMPs) [127]. Necrosis-elicited DAMPs releases are powerful activators of pro-inflammatory response, and a conspicuous feature of necrotic demise over apoptosis in situ is the infiltration of numerous phagocytic cells with early proinflammatory and cytotoxic phenotype. Notably, some molecular triggers of apoptosis promote the engulfment of apoptotic material by dendritic cells, antigen presentation and stimulation of a specific and further eliciting specific and moderate immunogenic responses [114,128].

More recently, biochemical classifications have been implemented, allowing the distinction between two apoptotic routes. Both pathways are mediated by proteases belonging to the caspase family. Molecular triggers of apoptosis can originate from either external (extrinsic apoptosis) or intracellular stimuli (intrinsic apoptosis). All routes culminate in the activation of effector caspase proteases (Caspase-3, -6 and -7), which will take an active part in apoptosis execution. Intrinsic apoptosis can be elicited by multiple stressing stimuli such as metabolic unbalance, hypoxia or DNA damage. In this case, apoptosis is initiated by mitochondrial outer membrane permeabilization (MOMP), under the positive or negative control of BCL2 family proteins, such as BAX, BAK, NOXA, PUMA, and BCL-2, BCL-XL SURVIVIN and MCL-1. Subsequently, mitochondrial rupture releases proteins such as cytochrome-c and SMAC which interact with APAF1 and pro-caspase-9 to form a cytosolic death protein complex called “apoptosome”. The tumour suppressor protein 53 (TP53;p53) is a transcription factor also capable of inducing intrinsic apoptosis, by promoting the transcription of BAX, PUMA, NOXA and BID, and repressing BCL-2 and, BCL-XL [129,130]. Extrinsic apoptosis is typically induced by the binding of ligands death receptors on plasma membrane such as FAS and TNFR1 and activate initiator caspase such as Caspase-8 and -10. Only non-inflammatory caspases are involved in apoptosis, while inflammatory types of caspases promote another regulated cell death pathway coming with necrotic-like features, known as pyroptosis [131].

Traditionally, apoptotic pathways have therefore been considered as the archetypal regulated cell death pathways (RCDs), in contrast with accidental cell death (ACDs), presenting typical necrotic features [114].

### 3.3. Necrosis: One Word That Can Mean Many Death Pathways… or No Pathway at All

Necrotic demise has historically been based on a set of morphological criteria: cellular and organelles swelling (aka “oncosis”), cytoplasmic granulation, and early plasma membrane leakage. Largely, necrosis has been considered as an “accidental” cell death pathway, passively occurring in response to physicochemical aggressions, leading to a dramatic alteration of cell homeostasis which is not supported by active cell machinery. More recently, multiple pieces of evidence have progressively challenged the dogma of necrotic demise as an unregulated cellular process. Several death pathways have now been identified, collectively referred to as regulated necrosis (RN) or non-apoptotic regulated cell death (RCD) [14]. A breakthrough in the field was provided by the identification of necroptosis in the lab of J. Yuan.

### 3.4. Necroptosis: A Backup Plan to Die When Apoptosis Fails

TNFα binding to its DR TNFR1 can elicit either the activation of the pro-survival pathway or cell death. While TNFα can elicit extrinsic apoptosis via caspase-8 activation, the use of pan-caspase inhibitors such as Z-VAD-FMK does not necessarily prevent cell death but leads to death with faster execution and necrotic morphology [132]. This TNF-elicited cell death occurring in caspase-compromised conditions can be prevented by Necrostatin-1 [133], and therefore demonstrates a non-apoptotic cellular pathway. As this necrotic form of RCD shares a part of its pathway with extrinsic apoptosis, it has been named by the neologism “necroptosis”. The anti-necroptotic activity of necrostatin-1 is attributed to the inhibition of the kinase activity of receptor-interacting protein kinase (RIPK)1 [134]. The central role of RIPK1 in response to cell stress was previously understood as a platform hub for integrating extrinsic stimuli, leading the cell to either activate the transcription of pro-inflammatory (and pro-survival genes via NF-κB and MAPK pathways activation) or igniting cell death [135]. Following TNFα binding to TNFR1, the scaffold property of RIPK1 supports the survival fate of the cell, harbouring proteins known as complex I, under the control of RIPK1 ubiquitinylation status. Preventing NF-κB and MAPK surviving pathways can be achieved experimentally by destabilizing complex I using antagonists for IAPs (also known as SMAC-mimetics) or by the inhibition of TAK1, therefore sensitising cells to TNF-elicited cell death [136]. If caspase-8 can be activated, the extrinsic pathway of apoptosis is initiated, culminating in apoptotic execution. If the caspase system is compromised by pharmacology or by a viral infection, cells expressing RIPK3 can initiate necroptosis by the formation of a RIPK1-RIPK3 complex named “necrosome”, via their RIP homotypic interaction motives, and forming an amyloidal protein complex. Among necrosome, cross-phosphorylation of RIPK1 and RIPK3 can be achieved at different sites which are not necessarily conserved in evolution [137,138,139], then recruits the pseudokinase MLKL by phosphorylation [140]. The RIPK3-driven phosphorylation of MLKL allows a change of conformation of p-MLKL, promoting its affinity to bind and oligomerize to phosphatidylinositol of the plasma membrane [141,142]. The oligomerization of p-MLKL compromises the integrity of the plasma membrane, leading to catastrophic osmotic disturbance and is therefore at the origin of the typical necrotic features of cells dead by necroptosis [143]. Beyond TNFα, or other ligands to DR can trigger necroptosis [144], such as IFNγ which can trigger necrosome formation via RIPK1, Jak1 and STAT1 [145]. Of note, necroptosis can be elicited in a RIPK1-independent way, using Toll-like receptors (TLR) 3 and 4 [146,147], or viral infections via acid nucleic sensors recruiting the RIPK3-MLKL axis [148].

### 3.5. Pyroptosis

In greek, “pyro” refers to “fire” and “ptosis” refers to “falling”. Pyroptotic demise was originally identified as a cell death that can eliminate immune cells and was deemed as apoptosis because of some aspects of its morphological and biochemical features, i.e., DNA damage, nuclear condensation, and the presence of apoptotic-like bodies [149]. Furthermore, this RCD shows positivity to markers of cell death previously recognized as typical for apoptosis, i.e., TUNEL staining, Annexin V positivity, PS exposure, and activation of the caspase system, even if the extent of each parameter can differ [150]. Importantly, pyroptosis distinguishes from apoptosis by the early permeability of plasma membrane and osmotic lysis, leading to a necrotic morphology, and partially responsible for its powerful inflammatory nature. While caspases involved in apoptosis are non-inflammatory, typical pyroptotic caspases belong to the inflammatory caspases and require Caspase-1, -4, -5, -11, culminating in the formation of membrane pores formed by Gasdermin-D. An interesting hallmark of pyroptotic demise is the formation of a protein platform called inflammasome, leading to the release of IL1ß and IL-18 by pores formed by Gasdermin-D, and participating in the powerful pro-inflammatory nature of this regulated form of necrosis [151]. Canonical and non-canonical pyroptosis can be triggered by various molecules such as ATP, toxins or bacteria. Pathogen-associated molecular patterns and danger-associated molecular patterns (PAMPs and DAMPs) commonly trigger pyroptosis by binding cytosolic pattern recognition receptors (PRRs) such as NLRP3. Downstream signalling pathways involve type I interferon generation and pro-inflammatory cytokines release [150,152,153]. Pyroptosis is now considered a therapeutic target in several forms of tumour-associated conditions [150].

### 3.6. CypD-Dependent Necrosis

Cell death elicited by the opening of mitochondrial permeability transition (mPT) has been identified in multiple pathological conditions involving ischemic injuries and inflammatory insults. It was often associated with apoptotic demise. However, growing evidence suggest that mPT opening generally promotes cell death with necrotic morphology [154,155]. The mPT pore belongs to the inner membrane of mitochondria and can be opened in response to elevated matrix Ca^2+^, and/or ROS. Cell death execution is mediated by the permeability of the inner mitochondrial membrane to small solutes, massive water supply and osmotic degradation of the cell. The exact composition of the pore is not clear however, there is consensus on the key role of peptidylprolyl isomerase F, also known as cyclophilin D (CypD) [117,156]. The genetic ablation of CypD or its pharmacologic inhibition delays cell demise [154,155].

## 4. Regulated Cell Death in Muscular Dystrophies

**Figure 1:**
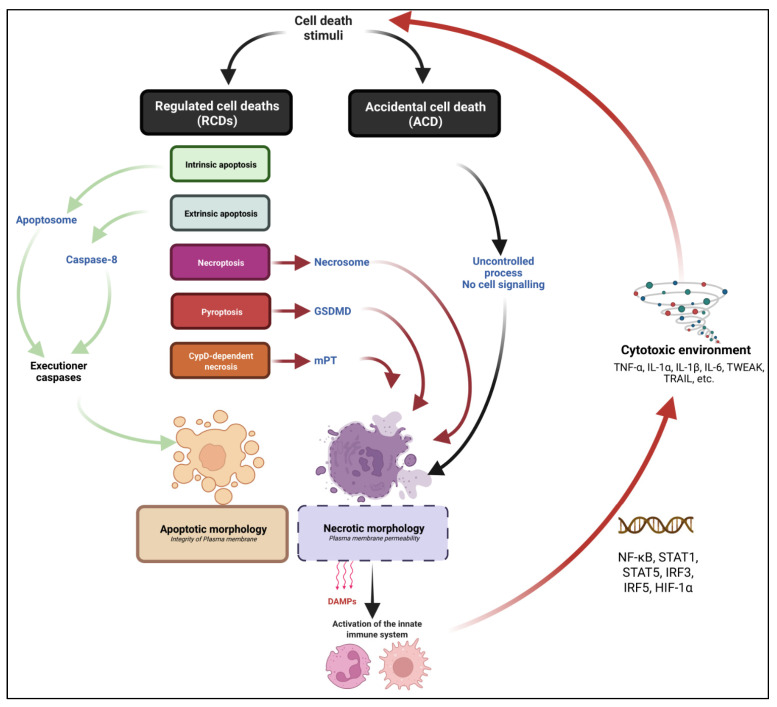
General view of the main cell death modalities with relevance in myology. Cell death can result from an accidental/unregulated or cell-regulated process. Accidental cell death (ACD) refers to a cell demise for which no identified signalling pathway is involved in cell death execution. Regulated cell death (RCD) refers to a cell death for which activation and execution are controlled, positively or negatively by a biochemical process. Cell demise can be genetically and/or pharmacologically prevented. RCDs pathways can lead to apoptotic or necrotic morphologies depending on the nature of the executioner machinery. Apoptosis: RCD which is controlled by non-inflammatory caspase proteases, culminating in a cell demise associated with typical morphology of cell rounding, nuclear condensation; membrane blebbing and apoptotic body formation. No early plasma membrane permeabilization is assessed. Two subtypes of apoptotic pathways are identified: intrinsic and extrinsic apoptosis, respectively mediated by the apoptosome formation and Caspase 8 activation. Necrosis refers to cell death morphology associated with early plasma membrane permeabilization. Necrotic death can be the consequence of an ACD or a non-apoptotic RCD. Necroptosis is a subtype of necrotic RCD. Necroptosis is genetically controlled by the RIPK1-RIPK3-MLKL axis. Pyroptosis refers to another subtype of necrotic RCD. Pyroptosis is controlled by non-apoptotic caspase and GSDMD pores formation. CypD-dependent necrosis refers to an RCD which is mediated by the formation of a mitochondrial permeability transfer pore (mPT), culminating in a necrotic demise. A typical consequence of necrotic morphology over apoptosis is the release of DAMPs, the infiltration and activation of myeloid infiltrate and the promotion of a cytotoxic environment including pro-inflammatory cytokines.

The emerging field of non-apoptotic forms of cell death including regulated necrosis has spread throughout the cancer, neurodegenerative disorders, and cardiovascular fields. Still, it takes time to update the scientific community on this conceptual revolution and apply it in all relevant tissues and disorders. In myology, there is a pressing need to reconsider previous conclusions as not all the latest updates in the cell death field have been integrated, possibly leading to legitimate misinterpretation of data. In publications dealing with muscle degeneration, the distinction between intrinsic and extrinsic is rarely acknowledged. Apoptosis used to be referred to in case of DNA fragmentation, or activation of MOMP, mPT, PARP-1, p53 or caspase-3, while it could also reflect early myogenesis, non-lytic cell stress or non-apoptotic RCDs such as pyroptosis, CypD-dependent necrosis, parthanatos, and necroptosis. Here, the main data dealing with RCDs in muscular dystrophies, integrating the understanding of the latest updates in the cell death field, will be summarized (Figure 1).

### 4.1. Apoptosis in MDs

Although, apoptotic demise is well-characterized in myogenic monocellular cells, its very existence in differentiated multinucleated cells is more controversial. Many studies reported that differentiated myotubes are dramatically more resistant to death upon apoptotic stimuli than MuSCs [157,158,159,160,161,162,163]. Still, the involvement of apoptosis has frequently been suggested in multiple muscular dystrophies, including dystrophinopathies [82,164,165], dystroglycanopathies [166,167], Calpainopathies [168], γ Sarcoglycanopathy [94], Collagen VI deficiency [169], merosinopathies [112,113,170], Oculopharyngeal muscular dystrophy [171] and FSHD [164,172,173,174]. Beyond the area of muscular dystrophies, evidence of apoptosis has been observed in sarcopenia caused by disuse or TNFα [175,176,177], and inflammatory myopathies [178,179]. Such evidence for apoptotic demise has mainly been provided by quantifying DNA fragmentation using TUNEL labelling. For some of these studies, caspase-3 activation and the upregulation of proteins associated with the machinery of intrinsic apoptosis, including p53 and BAX, were also considered as markers of apoptosis. Ca^2+^ intracellular concentrations are increased in mdx muscles [180], which control the activation of calcium-dependant proteases such as calpains [181,182]. Calpains can mediate the activation of the intrinsic apoptotic pathway. Furthermore, Ca^2+^-mediated mitochondrial permeability transition (mPT) promotes cytochrome *c* release and the activation of pro-apoptotic caspases [183]. Whether the expression of these proteins does necessarily demonstrate apoptotic cell death can legitimately be questioned. Indeed, muscle cells cope perfectly well with the activation of apoptotic caspases, which are required for myogenic differentiation [119,184]. Positivity to apoptotic markers should be taken with extreme caution since they are no longer considered specific for apoptosis by the scientific community working on cell death [116,117,184]. Interestingly, the validation of the apoptotic nature of degenerating muscle cells, and their role in pathogenesis has rarely been fully demonstrated by functional inhibition of apoptosis in vivo. The well-known non-apoptotic roles of caspases, especially in myogenesis should always be taken into consideration.

### 4.2. Necroptosis in MDs

**Figure 2:**
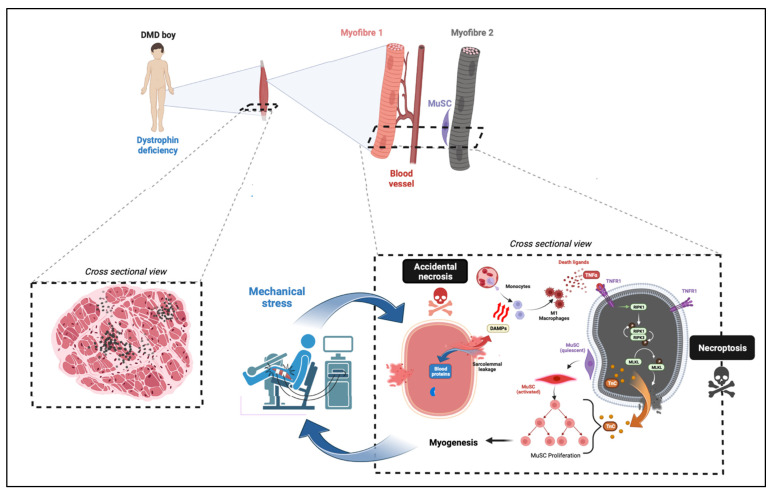
Model for necroptosis involvement in Duchenne muscular dystrophy. Limb muscles from DMD boys are affected by chronic necrosis affecting myofibres. Genuine milestone of cell death events is likely to come from exercised-induced breaks into sarcolemma missing dystrophin (necrosis by accidental process affecting Myofibre 1, pink colour). A direct consequence of this accidental necrosis is the activation of necroinflammation via the release of DAMPs, and the classical activation of infiltrated macrophages. M1 macrophages secrete pro-inflammatory molecules, including death ligands such as TNFα binding to their DR. TNFα binding to TNFR1 of myofibres and recruits RIPK1. The kinase activity of RIPK1 is required to recruit RIPK3 into the necrosome complex. RIPK3-dependent phosphorylation of MLKL promotes the recruitment of p-MLKL to the sarcolemma, the formation of pores, and compromises the integrity of the plasma membrane of myofibres (necrosis by necroptotic pathway). Myofibre necroptosis is associated with tenascine C (TnC) release, which supports MuSC proliferation and early myogenesis.

The first identification of a genetically programmed form of necrosis in the degeneration affecting skeletal muscle tissue was in dystrophinopathies [18] (Figure 2). In human DMD human biopsies, MLKL phosphorylation has been observed in myofibres, suggesting ongoing necroptotic demise in terminally differentiated muscle cells. Mouse and human dystrophin-deficient myofibres undergoing necrosis correlate with RIPK3 upregulation, which powerfully sensitizes muscle cells and cardiomyocytes to TNFα-elicited necroptosis [18,185]. RIPK3 depletion represses the necrotic spike affecting mdx mice at 3 weeks of age, indicating that the TNFα-mediated myonecrosis at the onset of mdx pathogenesis is due to necroptosis. This study does not report a significant role for RIPK3-dependent cell death in the mild degeneration occurring in adult mdx mice [18]. In dystrophin-deficient Golden retriever muscular dystrophy (GRMD) dogs, evidence of RIPK3-driven myonecrosis is also found in the diaphragm and the heart [186]. The long-term genetic ablation of RIPK3 in mdx mice dampens fibrosis deposition in respiratory and locomotor muscles. Another study showed that cardiotoxin muscle injury can be prevented by the inhibition of the kinase activity of RIPK1, or by the genetical depletion of MLKL [19]. Interestingly, myofibre necroptosis is associated with Tenascin-C release, which directly supports MuSCs proliferation and early myogenesis. Necroptotic demise could therefore be an important mechanism of myofibre death which paves the way for the early phases of muscle regeneration [19]. Long-term RIPK3 depletion does not show regenerative defects in mdx mice, suggesting that necroptosis is dispensable for long-term myogenesis and muscle remodelling in muscular dystrophy [186].

Beyond dystrophinopathies, the ectopic overexpression of DUX4, the poison protein causing muscle degeneration in FSHD triggers massive cell death in myoblasts and myotubes in vitro. Cell death cannot be prevented in caspase-compromised conditions, indicating a non-apoptotic mechanism [17]. However, DUX4-elicited myotube cell death can be prevented by RIPK1 and RIPK3 kinase inhibition. The ablation of RIPK3 in a mouse model for FSHD conditionally overexpressing DUX4 in muscle cells reduced CK levels and necroinflammation suggesting a role for necroptosis in mouse models of FSHD as well [17]. However, the relevance of DUX4-elicited necroptosis in FSHD in human remains to be confirmed. In polymyositis, few myofibres are immunoreactive to antibodies directed against RIPK3 and MLKL, and myonecrosis is reduced in a mouse model for C protein-induced myositis depleted for MLKL and RIPK3 [20]. RIPK1, RIPK3 and MLKL are also upregulated in degenerative muscles from Lipin-1-deficient mice, suggesting the activation of the canonical necroptosis [187]. However, this study does not provide functional validation of their findings by the prevention of necroptosis in vivo. Together, these reports indicate that necroptosis may be a common cell death mechanism to multiple degenerative neuromuscular conditions which involve chronic myofibre death.

Beyond the demise of differentiated myofibres, multiple neuromuscular disorders show dramatic cell death of other cell types such as motor neurons and cardiomyocytes. Cardiomyopathy and heart failure are central pathogenic features in the latest stages of DMD. RIPK1 phosphorylation (Ser166) has been found in the heart of GRMD dogs, indicating RIPK1 activation in dog cardiomyocytes. RIPK1 and RIPK3 are upregulated in DMD rats, which develop a significant cardiomyopathy [43,186]. Furthermore, RIPK3 depletion ameliorates cardiomyopathy in mdx mice, suggesting that necroptosis inhibition may have multiorgan benefits in DMD, tackling cell degeneration together with locomotor, respiratory and heart muscles [18,186]. Finally, the role of necroptosis has been identified in conditions associated with motor neuron degeneration such as spinal muscular atrophy, multiple sclerosis and ALS [188,189,190,191,192], even if its precise role has controversial aspects [193,194].

### 4.3. CypD-Dependent Necrosis in MDs

As mPT-driven cell death is regulated and highly dependent on mitochondria, it has been associated with apoptosis. In 2003, Irwin and colleagues identified that the phenotype of mice with Collagen VI deficiency improved with Cyclosporin A (CsA), a powerful immunosuppressor targeting T cells by inhibiting calcineurin in the calcineurin–phosphatase pathways [195]. CsA is also known as a potent inhibitor of mPT pore opening [169]. CypD deficiency or mPT inhibitors such as Cyclosporin A or Debio-025 improve muscle phenotype in small animal models of DMD (such as zebrafish and mice [196,197,198,199,200]) in Lama2 deficiency and δ-sarcoglycanopathy [200]. However, mPT inhibitors generates many detrimental side effects in vivo, including promoting carcinogenesis or reducing mitochondrial biogenesis and mitophagy [201,202,203,204].

### 4.4. Pyroptosis

So far, there is no clear demonstration of the existence of pyroptotic demise in myofibres, regardless of the pathologies affecting skeletal muscle. However, several interesting studies report the involvement of NLRP3-dependent inflammasome in muscle conditions and could, at least partially, be attributed to pyroptosis. NLRP3 is highly expressed in muscles from mdx mice [205,206]. Mdx*Nlrp3*^−/−^ mice show a decreased level of CK levels, establishing a link between NLRP3-dependent inflammasome and myofibre demise in dystrophinopathies [206]. NLRP3 and the NLRP3 adaptor ASC-1 are upregulated both in dysferlin- and dystrophin-deficient human muscles [104]. In dystrophic mouse models, other proteins associated with inflammasome activation such as caspase-1, IL-1ß are also upregulated. However, the depletion of ASC-1 failed to reduce myonecrosis. Whether the basal Ca^2+^ upregulation in dystrophic muscles can activate the NLRP3 inflammasome and generates myofibre cytotoxicity via the production of inflammatory cytokines is a hypothesis that remains to be addressed [207]. The inflammasome has also been found to upregulate and exert detrimental effects on muscle phenotype in muscles from VCP^R155H/+^ mice, a mouse model from Valosin-Containing Protein Myopathy [208].

## 5. Conclusions

### 5.1. Has Apoptosis Any Relevance in Myofibre Degeneration?

Considering the recent and extensive evolution of the cell death field today, there is clearly room for re-evaluation of data supporting the involvement of apoptosis, first by questioning the relevance and the lack of specificity of markers used to identify this RCD. Apoptotic demise has mainly been labelled in MD biopsies using TUNEL, and the expression of proteins belonging, directly or not to apoptosis [82,84,164,165,168,172]. However, since the 1990s, DNA fragmentation and positivity to TUNEL labelling are no longer considered specific for apoptosis [209]. TUNEL-positive nuclei are commonly found in non-apoptotic RCDs such as necroptosis, pyroptosis and parthanatos [210]. Similarly, PS exposure and Annexin V positivity are found in cells dying from regulated forms of necrosis [211]. Furthermore, the upregulation of apoptosis-related proteins does not necessarily represent a marker for cell demise. Caspase proteases can mediate non-apoptotic biological processes such as cell differentiation [212]. For instance, Caspase-3 -7 and -8 are required for myogenic differentiation [119,213]. Caspase-3 mediates the initiation of MuSC commitment by PAX7 cleavage which ends the self-renewal of MuSCs [214]. Therefore, the assessment of cleavage or upregulation of caspase-3 in skeletal muscle undergoing chronic degeneration/regeneration events could also be due to ongoing myogenesis, regardless of any apoptotic execution. The discrimination between regenerating fibres and dying fibres should thus be considered before concluding apoptotic death solely based on the activity of the caspase machinery. Likewise, proteins involved in MOMP such as BAX or BAK can also be associated with cell stress, regardless of the execution of intrinsic apoptosis [215]. Additionally, cell response to genotoxic stress requires p53, which stands at the crossroad between various physiological processes, cell death, and senescence [130].

While investigating apoptosis in degenerating muscles, another bias could be the nature of apoptotic-like nuclei. Indeed, a marker of myogenicity is not always used in combination with markers of cell death. In vivo, dystrophic muscles are largely infiltrated by waves of myeloid cells such as neutrophils, and macrophages before they undergo RCD, under the influence of efferocytosis [216,217,218]. Consequently, markers of the myogenic and differentiated status should therefore be associated with TUNEL labelling to avoid any confusion in the cell types undergoing DNA fragmentation. This comment also applies in vitro since myotube cultures are highly heterogenic in cell nature and a large percentage of nuclei are not necessarily included in terminally differentiated cells.

A key question about TUNEL labelling is rarely mentioned, but is crucial in the case of examining the phenotype of fully differentiated muscle cells: can DNA fragmentation really be interpreted as cell death in a syncytial cell structure? Myofibres (or large myotubes in vitro) are commonly formed by douzaines to hundreds of nuclei. The nuclear fragmentation, reflected by DNA fragmentation and/or substantial upregulation of caspase cleavage is catastrophic for cell homeostasis in mononucleated cells and therefore considered a point of no return, by strictly preventing the cell to restore homeostasis. However, this parameter is by nature strikingly different in a syncytial structure. Indeed, other nuclei of plurinucleated cells can compensate for the individual loss within the syncytial cell. The mean volume of myonuclei within myofibres can differ according to muscle activity [219], reflecting the plasticity of muscle fibres to cope with variations in nuclei density. While hyperplasia is a common phenomenon in response to mechanical load, loss of myonuclei in muscle disuse or ageing leads to muscle atrophy without myofibre degeneration [220,221]. In extraocular muscles, MuSCs are chronically activated, proliferate and fuse with existing myofibre, without signs of progressive hyperplasia, illustrating the importance of myonuclear turnover [222,223].

Therefore, whether studies assessing apoptosis in muscular dystrophies were true apoptotic evidence or not remains unclear. The role of apoptosis could theoretically be demonstrated by preventing executioner caspases in animal models of muscular dystrophies and quantifying the extent of remaining cell death. However, beyond the important role of caspase in myogenesis, preventing their activity has multiple side effects, and promotes alternative cell death such as necroptosis by increasing the stability of necrosome proteins [224,225]. A consensus on this subject is that (if apoptotic demise has a meaning in differentiated muscle cells), the role of apoptosis in myofibre death should be limited in MDs. Indeed, apoptotic-like myonuclei are relatively rare in degenerating dystrophic muscles [18,165]. Furthermore, typical consequence of apoptotic features since apoptosis does not compromise plasma membrane permeability, releasing sealed apoptotic bodies rather than DAMPs. MDs are characterized by an acute increase of DAMPs, CK levels, and inflammatory response to myofibre demise [21] illustrating response to necrosis and not apoptosis.

### 5.2. RCDs, New Therapeutic Targets for MDs?

Controlling pathogen infection and regulating the number of cells is required for tissue homeostasis, a process essential for normal development and physiology. However, cell death is also a common pathophysiological process in human health and controlling it would be a powerful means to reduce cell degenerating disorders. The discovery of apoptosis as a regulated form of cell death has made it possible to consider the possibility of developing therapies aimed at reducing excessive cell loss in pathology. Thus, targeting caspases for therapeutic purposes has been the subject of intense research [226], leading to promising results in cancer. However, caspase inhibition failed in the case of degenerative disorders. It has been speculated that a variety of reasons can prevent the efficiency of such therapeutic avenue. Firstly, certain key apoptotic caspases have non-apoptotic roles [227,228]. Another possible limiting factor is the interconnections of apoptotic machinery with other RCDs, making the inactivation of apoptosis ineffective for efficient prevention of cell demise [16]. Indeed, some caspases such as caspase-8 have paradoxical activities, promoting together apoptosis (via the cleavage of pro-caspase-3) and also cell survival (via the cleavage of RIPK1) [224]. Therefore pan-caspase inhibition prevents both apoptosis and promotes the formation of the necrosome, priming caspase-compromised cells to TNF-induced necroptosis [228]. Several clinical trials aiming at inhibiting necroptosis has been launched lately, and its efficacy, so far, is not yet strictly established. Several RCDs have been reported in degenerating muscles. This review focused on apoptosis, necroptosis, pyroptosis and mPT/CypD-dependent necrosis. Understanding RCDs in myology is in its infancy, however, other emerging forms of cell death might also participate in myonecrosis affecting MDs and remain to be identified. Intricate networks between RCDs suggest that some apoptotic or non-apoptotic cell death are complementary to each other and preventing one may have consequences on others. Therefore the complexity and pleiotropy of RCDs should be taken into consideration when evaluating the therapeutic activity of drugs in experimental disease models, and then in clinics [16].

## Data Availability

Not applicable.
